# Feeding Low- and High-Fibre Sunflower Meal to Broiler Chickens—Effects of Inclusion Rate and Age of Birds on the Production Traits, Carcass Composition, Nutrient Digestibility, Gut Viscosity, and Caecal Short-Chain Fatty Acid Content

**DOI:** 10.3390/ani16020162

**Published:** 2026-01-06

**Authors:** Kesete Goitom Tewelde, Brigitta Kiss, Tivadar Csiszér, László Pál, Nikoletta Such, Ádám Bartos, Károly Dublecz

**Affiliations:** 1 Institute of Physiology and Nutrition, Georgikon Campus, Hungarian University of Agriculture and Life Sciences, Deák Ferenc Street 16, 8360 Keszthely, Hungary; kesetehac@gmail.com (K.G.T.); brigitta.kiss@ubm.hu (B.K.); csiszer.tivadar@uni-mate.hu (T.C.); pal.laszlo@uni-mate.hu (L.P.); such.nikoletta.amanda@uni-mate.hu (N.S.); bartos.adam.sandor@uni-mate.hu (Á.B.); 2 Department of Animal Sciences, Hamelmalo Agricultural College, National Higher Education and Research Institute, Keren P.O. Box 397, Eritrea

**Keywords:** sunflower meal, broiler chicken, production traits, carcass composition, nutrient digestibility, nitrogen excretion, gut viscosity

## Abstract

Sunflower meal (SM) is an economically important, inexpensive, and locally abundant alternative protein source in the Eurozone. The study aimed at investigating the effects of feeding two forms of SM on the production traits, carcass composition, nutrient digestibility, and some gut parameters of broiler chickens. A total of 600-day-old Ross 308 male broilers were fed five isocaloric and isonitrogenous diets. Besides the control diet (C), the high- and low-fibre SMs (HFSM and LFSM) were fed at 20% (HFSM20 and LFSM20) and 30% (HFSM30 and LFSM30). Compared to the C, feeding the SM-containing diets did not affect the feed intake (FI) of birds. In the finisher phase, the HFSM30 treatment resulted in significantly higher, while the LFSM30 diet in significantly lower body weight gain. All SM treatments impaired FCR, but the difference was significant only in the grower phase. In comparison with the C, the SM treatments failed to modify carcass composition. The fat digestibility and the AMEn content increased, while the starch digestibility decreased when SM was fed. Except the LFSM30 treatment in the grower phase, the Nitrogen retention of birds was not affected. The SM-containing diets reduced the urinary N excretion, and the total N excretion of growers. Furthermore, the HFSM30 reduced the jejunal viscosity during the grower and finisher phases. The treatments did not modify the short-chain fatty acid contents of the caeca. In conclusion, SM can be used even at 20 and 30% in the nutrition of broiler chickens. However, the responses are affected by the age and the fibre content of SM.

## Simple Summary

The demand for poultry products is increasing in line with the rapid increase in the world’s population. It also means that more cereal grains and protein sources are needed for human nutrition, and more industrial by-products will be used in animal nutrition. Sunflower meal (SM), the by-product of the oil industry, is available in high amounts. However, its high fibre content is a limiting factor. In the frame of this work, a high- and a low-fibre SM was fed with broiler chickens at two inclusion rates, and the responses of animals were evaluated. The main conclusions are that the responses of feeding SM depend on the inclusion rate (20 or 30%), the fibre content of SM, the age of chickens (grower phase or finisher phase), and the investigated parameter. Surprisingly, the body weight gain (BWG) of the younger animals was not affected by SM, but in the grower phase, the highest BWG was registered in the 30% high-fibre SM group, and the lowest gain belonged to the 30% low-fibre treatment. SM diets increased the digestibility of fats but decreased the starch digestion. The SM-containing diets resulted in lower urinary nitrogen excretion, which can lead to reduced ammonia emission. The carcass composition and the short-chain fatty acid composition of the caeca were not affected. Our findings revealed that SM can be used as broiler feed even at 30% without compromising the production parameters.

## 1. Introduction

Food demand and food value chain globalisation are increasing very fast; at the same time, numerous environmental factors are continuously influencing crop productivity and stability. This necessitates the practice of a sustainable poultry production system through the use of locally available, cheaper low-protein diets [1]. Globally, poultry meat consumption is projected to grow by about 21% and reach 173 Mt of ready-to-cook meat, which accounts for 62% of additional meat consumption. This will provide 45% of the protein consumed from all meat sources by 2034 [2]. Climate change impacts agricultural yields through global warming, extreme weather events, and changes in precipitation patterns. This leads to reduced yield, lower affordability, and price instability. It also affects global trade, especially for imported crops and animal feed, which in turn affects food security [3]. This indicates that food and feed market instability is increasing, mainly due to climate change, which reduces crop production prediction [2].

In chicks, feed cost accounts for about 70% of the total production cost [4,5]. A recent study reported that, over a four-year production period, the average feed cost accounts for 71.9% (69.6 to 76.1%) of the total production cost [6]. Feed cost and the price of both egg and meat increase in parallel; therefore, the higher the feed cost, the more expensive and less affordable poultry products become for most people worldwide. Protein is the most expensive feed component, as a result, feed manufacturing industries, poultry farmers, and researchers are looking for locally available, cheaper alternative protein sources in order to replace maize and soybean in ration formulations, a better approach to overcome increasing feed cost. However, the feed manufacturing companies could not find an alternative that could fully replace soybean meal. As a result, many countries, including EU member countries, are highly dependent on imported protein sources, mainly soybean meal [7]. Peng et al. [8] highlighted that global soybean trade has rapidly expanded, the trade dynamics are driven by climate, and the expansion has caused deforestation, biodiversity loss, and food security challenges. In addition, Hamed et al. [9] found that one third of the global soybean production deficit in 2012 was attributable to climate change. Therefore, climate change and soybean importation are the root cause for the continuous price increment in poultry feed, insecurity, and high carbon footprint [10,11].

The increase in demand for soybean in the global market has gained an increasing importance, in Brazil. This leads to the expansion of cultivable land to increase the production of soybean through clearing forests. Its expansion is a big threat to climate change resulting from deforestation [12]. Therefore, it is obvious that the reliance on a single protein source (soybean) may cause availability, production cost, and environmental problems, which are currently forcing researchers and nutrition experts to search for alternatives [13]. Possible alternatives are legume seeds, extracted meals, dried distilled grains with solubles, meat meals, fish meal, insect meal, and seaweeds. The challenge with such feed ingredients is that there is no clearly determined information regarding their maximum inclusion rates [11]. Such challenges are evident in optimising sunflower meal (SM) inclusion, mitigating the knowledge gap and inconsistencies in including SM in broiler diets [14]. Therefore, to ensure sustainable broiler production and reduce the environmental impact of meat production the use of locally available cheaper protein diets and reducing soybean importation is timely needed [15]. This can be mitigated by identifying and incorporating locally available alternatives in broiler feed formulation, a subject of growing interest to reduce dependency on traditional sources and improve sustainability. In this case SM could be one of the potential alternatives.

Sunflower is a widely cultivated, important oil crop that ranks third in oilseed and seed-meal production after soybean and rapeseed meal, as well as fourth among the vegetable oils such as palm, soybean, and rapeseed oils [16,17,18]. This indicates that it is both competitive and abundantly available [16]. In addition, the advantages of using SM as an alternative protein source include a lack of antinutritional factors, relatively low cost, richness in sulphur-containing amino acids, and enough crude protein (CP) and essential amino acids. The only limitations are its high fibre, lysine and threonine deficiencies, low metabolisable energy, and the polyphenolic compounds such as chlorogenic contents when compared with soybean meal [10,19].

This can be solved through a careful feed formulation [14]. According to different reported feed-analysis results, the dietary fibre of SM ranges from 12% to 25% [20,21,22]. The difference in chemical composition is rooted in varied processing methods, such as the extent of dehulling and oil extraction efficiency. As a result, the oil industry produces three types of SM, such as undehulled (28 to 30% protein and 25% fibre), partially dehulled (33 to 35% protein and 21% fibre), and double dehulled (38 to 40% protein and 15% fibre). The partially dehulled type is the most widely available [22]. The price varies depending on region and type of SM. The EU average price in 2025 was 218.8 €/ton (ranging from 170.86 € to 312.37 €/ton) [23]. SM is also rich in essential fatty acids and can be included in the broiler diet to enrich the omega-3 and omega-6 polyunsaturated fatty acid content of poultry products [24].

Mbukwane et al. [14] concluded that dehulled sunflower oilcake can be incorporated in finisher and post finisher diets up to 13.5% without negatively affecting the FI, body weight gain (BWG), and feed conversion ratio (FCR). Substituting soybean meal with SM at 3%, 6%, and 9% did not have a significant influence on growth performance and carcass characteristics in broilers fed the diet from 8 to 21 days, 22 to 35 days, and 8 to 35 days [25]. Replacing soybean meal with high-protein SM at 5% to 15%, 10% to 25%, and 15% to 26.5% in the starter (10 d), grower (24 d), and finisher (49 d) phases, respectively, did not show a significant difference in growth performance and FCR in all age groups. High-protein SM can replace up to 53% of the dietary CP. The safe inclusion of high-protein SM in order to achieve a higher performance is 10%, 20%, and 23% in the starter, grower, and finisher periods, respectively [26]. However, the safe inclusion rate varies with the type of SM used, for instance, the safe inclusion rate in the case of sunflower cake is 10% during the finisher phase from 21 to 42 days of age [27]. Replacement of soybean meal with SM at 0%, 10%, and 20% increased digesta viscosity significantly with increasing levels of SM [28]. SM that contained 29.1% crude fibre (CF), was included at 0%, 5%, and 6% during the starter phase (1–21 days) and at 0%, 8%, 10% during the grower or finisher phases (22–42 days). These were fed ad libitum and had no effect on jejunal digesta viscosity [29]. SM with 18.5% CF was fed at a rate of 16% to pullets and 20% to laying hens, which significantly reduced jejunal viscosity compared to the control diet. Jejunal viscosity was also significantly higher in laying hens than pullets [30]. SM can be used as an energy source for broilers. The apparent metabolisable energy (AME) and its corrected version for zero N-retention (AMEn) content of SM varies with its fibre content and processing methods. Azam et al. reported that the AME of SM ranges from 11.2 to 11.25 MJ/kg [31] and the total AMEn is up to 9.5 MJ/kg [32]. There is no research directly comparing low-fibre and high-fibre sunflower mean in broiler-grower and broiler-finisher phases on the caecal SCFAs, digestibility of AMEn and excreta-N composition. As per the knowledge of the authors, there is also no research that includes low-fibre SM (LFSM) and high-fibre SM (HFSM) at up to 30% inclusion rates.

The inclusion of SM in the broiler diet mixed along with other low-protein diets using a 15% combination of corn gluten meal (2.5%), dried distillers’ grain (7.5%), and SM (7.5%) compared with the control diet after formulating isonitrogenous and isocaloric diet failed to affect the total excreta-N composition [1]. Other findings revealed that feeding low-protein diets or reducing the protein content of the broiler ration resulted in reduced N excretion [33,34,35]. In addition, reducing dietary protein with a balanced amino acid supply does not affect dietary AMEn, it even improves nitrogen retention and energy utilisation [33,34].

The above-mentioned characteristics of SM make it a better alternative to minimise the dependency on the traditional crops in chicken production so as to reduce production cost and the environmental impact of importing soybean meal far from Latin America. Examining the use of different levels of SM in broiler diet formulation on production performance, carcass traits, nutrient digestibility, nitrogen excretion, viscosity, and short-chain fatty acid (SCFA) is currently a hot topic to determine its effect and the possible maximum inclusion rate in broiler diet. Though limited information is available on the maximum inclusion levels of SM in broiler diets, Such et al. [36] reported that SM can be used up to 30% in the diets of pullets and laying hens. They also concluded SM could entirely replace soybean meal.

If pullets can tolerate SM inclusion up to 30%, then there is a potential to include SM in the broiler diet as well. As a result, the objective of the current study was to investigate the inclusion of both HFSM and LFSM up to 30% level in the broiler diet and its impact on production performance, carcass traits, N retention, excreta N contents, caecal short-chain fatty acids, and digesta viscosity, and to obtain more information on the maximum inclusion rate.

## 2. Materials and Methods

### 2.1. Birds and Housing

A floor pen trial was carried out at the experimental farm of the Department of Animal Nutrition and Nutrition Physiology, Georgikon Campus, Hungarian University of Agriculture and Life Sciences. The campus ethics committee approved it in accordance with animal welfare legislation under the licence number MAB-1/2023. A total of 624-day old male broiler chicks (Ross 308) with a body weight of 45.6 ± 0.4 g were purchased from a local hatchery (Gallus Ltd., Devecser, Hungary). Then 600 birds were randomly allocated to 25 experimental pens. There were five treatments with 120 chickens in each treatment. Each treatment had five replicates each containing 24 birds. Each pen was provided with clean wood shavings as a bedding material, and an adequate area for exercise (14 birds/m^2^) within an environmentally computer controlled illuminated house. As a result, the environmental conditions of the different pens within the house such as heating, lighting, and ventilation were identical. Birds in each pen shared the same feed and water as a group. A balanced feed and automatic drinking water were supplied ad lib.

The lighting programme was set according to the guidelines given by Aviagen for Ross 308 [37]. Birds received 24, 23, 20, and 18 lighting hours on day 0, days 1 to 7, days 8 to 11, and days 12 to 38, respectively. Each bird was vaccinated against infectious bronchitis (CEVAC BRON), Newcastle disease (VITAPEST), and infectious bursal disease (CEVAC TRANSMUNE) in the hatchery. The vaccine is produced by Ceva (Ceva Sante Animale, Libourne, France).

### 2.2. Experimental Diets

The nutrient contents of HFSM and LFSM are shown in Table 1. HFSM contained less protein, amino acids, but more structural and insoluble fibre. On the other hand, LFSM contained more protein, amino acids, and less insoluble fibre. The soluble fibre fraction of LFSM was higher than that of HFSM.

The composition of the experimental diets is shown in Table 2. Feed was formulated according to the guidelines of the breeder company [37]. Five isocaloric and isonitrogenous diets were prepared. The five treatments consisted of a soybean-wheat-corn based control diet (C), and diets that contained HFSM and LFSM at inclusion levels of 20% (HFSM20, LFSM20) and 30% (HFSM30, LFSM30); these were fed in the grower (days 11–24) and finisher (days 25–38) phases. Diets in the starter phase (day 0–10) did not contain SM. The two forms of SM were incorporated mainly at the expense of soybean meal, and the lower AMEn content of SM was compensated for with more sunflower oil (Table 2). The measured nutrient content of the diets shows that the values were close to the predicted ones (Table 3).

### 2.3. Chemical Analysis

The proximate nutrients of the experimental diets were analysed with well-known standard methods. The neutral detergent fibre (NDF) was determined sequentially as described by Van Soest et al. [38] and expressed on Ash free basis. The gross energy (GE) of the diets and excreta samples was analysed using a bomb calorimeter (IKA C6000, IKA-Werke GmbH & Co., Breisgau, Germany). The starch content was measured by the polarimetric method according to European Directive 152/2009 [39]. The amino acid content of the diets was analysed using an automatic amino acid analyser (Ingos Amino Acid Analyzer AAA 400, INGOS s.r.o., Prague, Czech Republic) according to the ISO 13903:2005 standard [40]. The titanium dioxide (TiO_2_) determination was carried out by an UV spectrophotometer (Jenway 6100, Bibby Scientific Limited, Staffordshire, UK), with absorbance measurements taken at a wavelength of 410 nm, according to Short et al. [41].

### 2.4. Measurements and Samplings

Production parameters such as the FI and body weight (BW) of all chickens were measured at the end of the grower (day 24) and finisher (day 38) phases on pen basis. Body weight gain (BWG) was then calculated by deducting the initial average BW from the final average BW. FCR was calculated based on grams of feed consumed to produce a gram weight gain. An indigestible marker, TiO_2,_ was mixed at a rate of 5 g/kg in the grower and finisher diets. On day 24, 16 chickens from each treatment group were selected randomly and placed into 40 balance cages in pairs, representing eight replicates of each treatment. Again, on day 38 a total of 40 chickens, 8 birds per treatment, were also assigned to balanced cages for carrying out the digestibility trial. Each cage was equipped with automatic drinkers and feeders. After 2 days of adaptation (on days 26 and 27, and days 40 and 41) representative excreta samples were collected, avoiding contamination with feed and feathers. The daily samples were stored in a refrigerator. On the second day the daily samples were homogenised, and around 50 g of excreta were frozen and stored until further analysis. On days 27 and 41, all the animals of the digestibility trial were euthanised and slaughtered in compliance with the animal welfare legislation (Hungarian Government Decree 40/2013). The abdominal cavity of the animals was opened immediately and the digesta of the whole jejunum and the caecal content of the left sacks was collected. The gut contents were gently squeezed and poured into Eppendorf tubes (0.5 mL) and stored in a refrigerator at −20 °C prior to viscosity and SCFA analyses. After the grower phase, the gut contents of two chickens from the same cage were pooled. After the collection of gut contents, the carcass composition of chickens was also determined. The following parameters were measured: carcass weight (weight without legs, head, intestine, skin and feathers), deboned breast meat, abdominal fat, and thighs.

### 2.5. Digestibility and Metabolisable Energy Calculations

The following equation was applied for the faecal digestibility calculations:

$$\mathrm{Nutr}.\;\mathrm{digestibility}\;=\left(\;\frac{{\mathrm{Nutr}.}_{\mathrm{diet}}-({\mathrm{Nutr}.}_{\mathrm{excreta}}({\mathrm{TiO}}_{2\;\mathrm{diet}}/{\mathrm{TiO}}_{2\;\mathrm{excreta}}))}{{\mathrm{Nutr}.}_{\mathrm{diet}}}\right)\times 100$$

where

Nutr. digestibility = nutrient digestibility (%);
Nutr._diet_ = nutrient content of the diet (g/kg);
Nutr._excreta_ = nutrient content of the excreta (g/kg);
TiO_2 diet_ = TiO_2_ content of the diet (g/kg);
TiO_2 excreta_ = TiO_2_ content of the excreta (g/kg).

$$\mathrm{AME}={\mathrm{GE}}_{\mathrm{diet}}-({\mathrm{GE}}_{\mathrm{excreta}}\times \left(\;\frac{{\mathrm{TiO}}_{2\;\mathrm{diet}}}{{\mathrm{TiO}}_{2\;\mathrm{excretra}}}\right)$$

where

AME = apparent metabolisable energy (KJ/g);
GE _diet_ = gross energy content of the diet (KJ/g);
GE _excreta_ = gross energy content of the excreta (KJ/g);
TiO_2 diet_ = TiO_2_ content of the diet (g/kg);
TiO_2 excreta_ = TiO_2_ content of the excreta (g/kg).

$$\mathrm{Nitrogen}\;\mathrm{ret}.=\left[\frac{N_{\mathrm{diet}}-(N_{\mathrm{excreta}}\times ({\mathrm{TiO}}_{2\mathrm{diet}}/{\mathrm{TiO}}_{2\;\mathrm{excreta}}))}{N_{\mathrm{diet}}}\right]\times 100$$

where

Nitrogen ret. = nitrogen retention (%);
N_diet_ = nitrogen content of the diet (g/kg);
N_excreta_ = nitrogen content of the excreta (g/kg);
TiO_2 diet_ = TiO_2_ content of the diet (g/kg);
TiO_2 excreta_ = TiO_2_ content of the excreta (g/kg).

$$\mathrm{AMEn}\;\left(\mathrm{kJ}/\mathrm{kg}\right)=\mathrm{AME}-\left(N_{\mathrm{retention}}\times 34.4\right)$$

where

AME_n_ = apparent metabolisable energy nitrogen corrected zero N retention;
AME = apparent metabolisable energy (KJ/g);
N_retention_ = nitrogen retention (gN/gdiet);
34.4 = constant, the GE value of uric acid (KJ/g N).

### 2.6. Viscosity Measurement

Stored samples were thawed overnight, centrifuged using a Thermo Scientific autoclavable 138 °C Heraeus Megafuge 16r centrifuge at 10,000× *g* RPM speed and 25 °C adjusted temperature for 10 min (Thermo Fisher Scientific, Waltham, MA, USA). Samples were centrifuged after every three-sample analysis. After centrifuging, 0.5 mL supernatant was taken using an adjustable volume pipette-man and poured into the cup. The sample-containing cup was then assembled with the adjustment ring and fitted with the cone spindle for viscosity analysis. Viscosity analysis was conducted with a programable digital Wells-Brookfield LVDV-II+Pro Cone/Plate Viscometer (Brookfield Engineering, Waltham, MA, USA) with LCD display output to connect a PC. For reading the results with ±1% accuracy range and ±0.2% repeatability a special software was used (DV-Loader V2.1). The viscometer is connected to a temperature bath to maintain temperature at 25 °C for precise and reproducible viscosity measurement.

### 2.7. Determination of Total N, NH_4_-N, and Uric Acid-N Contents of the Excreta Samples

From the excreta samples their dry matter (DM), total N, NH_4_-N, and uric acid-N contents were also determined. Total N was analysed using Kjeldahl method using Foss-Kjeltec 8400 analyser unit (Nils Foss Alle 1, Hilleroed, Denmark). NH_4_-N and uric acid-N were measured according to Peter et al. [42] and Marquardt et al. [43], respectively. The urinary N content was calculated as the sum of the NH_4_-N + uric acid N. The faecal N content was calculated as the difference between total N and urinary N.

### 2.8. Short-Chain Fatty Acid (SCFA) Determination

After euthanizing the birds, cecum chyme samples were collected for SCFA analysis and immediately stored at −20 °C until analysis. Samples were thawed and prepared for gas chromatographic SCFA determination according to Atteh et al. [44]. Frozen samples were mixed and thawed thoroughly. Then, 250 µL of the digesta was mixed with 600 µL of 1.11 M HCl. The gas chromatograph, equipped with a 30 m (0.25 mm 178 i.d.) fused silica Nukol column (Supelco Inc., Bellefonte, PA, USA), used a flame ionisation detector with a 1:50 split injector. The injector volume was 1 µL at 220 °C, and detection occurred at 250 °C. Helium was used as a carrier gas at a pressure of 83 kPa. Calibration was performed using standard SCFA mixtures (1, 4, 8, and 20 mM) of acetate, propionate, n-butyrate, and n-valerate.

### 2.9. Statistical Analysis

All statistical analyses were carried out by the software package SPSS 29.0 for Windows, (IBM Corp. in Armonk, NY. USA), using a randomised block design. The pens were the experimental units. The averages of tested parameters were analysed by one-way analysis of variance (ANOVA). Normality and homogeneity of variances were tested by Shapiro–Wilk and Levene test, respectively. Significant differences between groups were tested by Tukey HSD test and if the distribution of data was not homogenous, Games-Howel and Welch tests were applied. Statistical significance has been declared at *p* < 0.05.

## 3. Results

### 3.1. Production Traits

The production performance of broilers is shown in Table 4. No significant difference was observed in FI (*p* = 0.375) and BWG (*p* = 0.719) during the grower and finisher phases among all treatment groups. Unexpectedly, significantly higher BWG (*p* = 0.023) was recorded in the finisher phase at the HFSM30 treatment. Feeding the LFSM decreased the weight gain of the birds at 20% numerically, but at 30% significantly, compared with the control. In the grower phase both the HFSM and LFSM diets resulted in significantly higher FCR (*p* = 0.001) compared with control. The same tendency was found in the finisher phase, but in this case only the LFSM20 treatment caused significantly FCR higher (*p* = 0.036).

### 3.2. Effects of Dietary Treatments on Carcass Composition

Table 5 shows that the SM treatments did not affect the relative carcass (*p* = 0.289), breast meat (*p* = 0.083), and thigh weights (*p* = 0.479). The abdominal fat percentage, however, increased when the LFSM-containing diets were fed. The difference between the LFSM20 and HFSM30 groups was significant (*p* = 0.030). The abdominal fat percentage of birds fed LFSM20 was 54.24% higher than the birds fed HFSM30.

### 3.3. Nutrient Digestibility and Metabolisable Energy Measurements

The treatments resulted in nutrient dependent changes in the digestibility. LFSM at both inclusion rates improved the absorption of crude fat, at *p* < 0.001, after the grower phase (Table 6). On the other hand, all SM-containing diets decreased the digestibility of starch (*p* < 0.001) in comparison with the control. The lowest starch digestion belonged to the HFSM30 group. The treatments affected AME in line with the digestibility of fats. The LFSM diet at 30% inclusion rate increased AMEn (*p* < 0.001) by 0.7 KJ/g. The nitrogen retention of chickens was affected only by the treatment LFSM30 (*p* < 0.001) during the grower phase. This diet resulted in significantly higher N retention in comparison with the control and HFSM groups. After the finisher phase, at day 41, the highest and lowest fat digestibility was recorded in the case of the LFSM30 and HFSM20 diets (*p* < 0.001), respectively. The starch digestion was also affected negatively by SM diets at this age. The differences were significant for the HFSM30 and LFSM30 treatments (*p* < 0.001). The N retention of chickens did not change in the older birds (*p* = 0.424).

### 3.4. Effects of Dietary Treatments on the Dry Matter Content and Nitrogen Forms of Excreta

According to the findings of the current study, the inclusion of HFSM and LFSM at 30%, reduced the total N excretion of chickens significantly (*p* = 0.028) at day 27 (Table 7). Both the NH_4_-N (*p* = 0.001) and uric acid-N (*p* = 0.004) excretion declined in the SM groups, resulting in a significant decrease in the urinary N excretion in the groups of HFSM20, HFSM30, and LFSM20 (*p* = 0.002). No significant differences in these parameters were found after the finisher phase, at day 41.

### 3.5. The Jejunal Digesta Viscosity and Caecal SCFA Results

Although SM contains soluble fibre fractions, none of the HFSM and LFSM diets increased the viscosity of the jejunal gut contents in comparison with the control diet (Table 8). Interestingly, feeding HFSM at 30% resulted in the lowest viscosity (*p* < 0.001) at both age groups. The age and feed interaction effect on digesta viscosity showed that growers had a significantly lower viscosity (*p* = 0.005) an indicated that viscosity increases with age.

The SCFA content of the caeca was not influenced by the SM treatments (Table 9).

## 4. Discussion

### 4.1. Production Traits

Several studies have been conducted on the effect of feeding SM to broiler chickens [4,14,19,25,45,46,47,48,49,50]. Some findings revealed that dehulled sunflower oilcake can be included in the broiler diet up to 13.5% [14], substituting for soybean meal up to 9% [25], and a low-fibre, high-protein SM with 45.4% CP replacing soybean meal up to 15%, 25%, and 26.5% at days 10, 24, and day 49 [26], respectively, had no negative effect on production traits. The safe inclusion rate for better performance was achieved at 10%, 20%, and 23% in the starter, grower, and finisher phases, respectively. SM at 15% and 20% inclusion rate did not affect WG, FI, and FCR, however, at 25% deteriorates FCR [48]. Other findings reported that SM with 36% CP included at 10% had no effect on average BW, daily gain, average FI, and FCR on days 21 and 35 [51]. Broilers given diets containing 0%, 4%, 8%, and 12% SM had similar BW, FI, and FCR [52]. Based on their effects on the production parameters these research findings were only partly in line with the current study. In our case, the responses depended on the fibre content of SM and on the investigated parameter. HFSM, for example, tended to increase the FI of birds but, surprisingly, also resulted in higher BWG at 30%. This positive effect of HFSM was more pronounced in the finisher phase and could be related to the more developed digestive system in the finisher phase, as broilers can tolerate higher fibre at this age. Furthermore, the fibre of HFSM in older chickens can stimulate the gizzard, which might help to reduce viscosity in the small intestine and increase gut motility and the digestion of broiler chickens [46]. Since both FI and BWG increased, HFSM did not modify the FCR in comparison with the control treatment. In our case, LFSM did not affect the FI, however, it impaired the BWG and FCR of chickens. This result was not expected since the diets were isocaloric and isonitrogenous; the reason for the negative effect of LFSM is unknown.

There are also some research outcomes with contradicting results [13,27,29,50,53]. Replacing soybean meal with dehulled SM in the wheat-corn-soybean based diet fed to non-sexed Rose 308 birds had no effect at up to 30% inclusion level. However, the substitution at 60% (19% of the diet) and 100% (28.2% of the diet) levels caused a linear reduction in BW and FI [13]. Sunflower cake was fed to Cobb 500 birds at a rate of 0%, 5%, 10%, 15%, and 20% both at the age of 1 to 21 days [27,53] and 22 to 42 days [27]. Their findings revealed that at 15% and 20% inclusions the final BW, WG, and FI linearly decreased with increasing levels of sunflower cake. There were slight differences in birds’ type, age, sex, sources of energy, feed ingredients used for feed formulation, the type of SM used, and inclusion rates between the above-mentioned studies and our trial, which could be the reason for the contradicting results. In addition, the higher amount of sunflower oil added to the HFSM- and LFSM-containing diet might also affect the production traits. Findings revealed sunflower oil is rich in flavonoids—an antioxidant that reduces the oxidative stress, improves immune function, stimulates intestinal tract functions, enhances digestive secretions, nutrient absorption, and metabolism, thereby improving growth due to the bioactive compounds in the oil that support intestinal health, boost digestion, and scavenge free radicals [45,54]. To evaluate the fibre and fat effects separately warrants further investigation.

### 4.2. Carcass Yield and Abdominal Fat Percentage

The SM treatments did not show a significant difference in carcass yield, carcass cuts, and the ratio of the meat parts. Significantly higher abdominal fat was observed only between the groups fed LFSM20 than HFSM30. Berwanger et al. [27] reported also that sunflower cake does not significantly affect the carcass cuts and abdominal fat percentage up to 20% inclusion and carcass yield up to 15%. Similarly, SM up to 20% did not have a significant difference in carcass weight and carcass cuts [10]; likewise, the inclusion of SM at 4%, 8%, and 12% did not affect carcass characteristics on day 42 [52]. SM at 0%, 5%, 6%, 8%, and 10% did not affect jejunal digesta viscosity, carcass characteristics, breast meat yield, and abdominal fat [29]. Sunflower inclusion at 6% and 8% in the growers’ stage, and at 10% and 16% during the finisher stage had no effect on carcass traits [50]. When SM was included up to 10% in the broiler diets, no difference was observed in dressing percentage [51]. Sunflower cake failed to affect carcass yield and abdominal fat up to 10% inclusion and even did not affect breast and thigh-plus-drumstick when increased to 20% compared with the control [53].

These findings align with the present study, where the inclusion of HFSM and LFSM up to 30% had no effect on carcass weight, breast fillet, thigh-plus-leg weight, or abdominal fat percentages compared to the control diet. However, it is important to note that, though all these findings agree with the present study, their inclusion rates were less than 30%. Therefore, based on the current study results, it can be concluded that SM has only limited effect on carcass traits and abdominal fat percentage, if the diets are isocaloric and balanced in essential amino acids.

### 4.3. Nutrient Digestibility, Metabolisable Energy, and Nitrogen Excretion

Compared to the control, only LFSM caused significant difference in this trial. The low-fibre SM increased significantly the faecal digestibility of fats and AMEn at both age categories. However, LFSM significantly decreased starch digestibility (day 27) and only at 30% (day 41). The N retention of birds increased in the LFSM group only after the grower phase.

The traditional HFSM failed to modify fat digestibility, AMEn and N retention at day 27, however, decreased the digestion of starch. After the finisher phase 20%, HFSM decreased fat digestibility and decreased starch digestion at both inclusion rates but did not modify AMEn and N retention in comparison with the control treatment. The results suggest a negative correlation between the fibre intake and starch digestibility, which could be the results of the well-known diluting effects of fibre, which hinders the efficiency of α-amylase. The reason for the opposite trend in fat digestion could be that fibre does not significantly disturb micelle formation in the small intestine [55]. However, the improvement over the control is hard to explain.

Karkelanov et al. [56] fed broiler chickens three types of SMs that vary in the degree of dehulling, resulting in CP contents of 37.4%, 40%, and 42.5% and corresponding CF contents of 21.7%, 20.2%, and 17.2%, respectively. These meals were included in the diet at a rate of 20% from day 8 to day 21. No significant differences in AME were observed between the first two SM types; however, the third high-protein, low-fibre SM significantly increased AME. These results can be compared with our HFSM and LFSM results at day 27, where HFSM did not affect AME digestibility, whereas LFSM significantly improved AME digestibility. In the trial of Bilal et al. [48] SM at 15%, 20%, and 25% levels in the broiler diet linearly improved crude fibre digestibility but failed to affect DM, CP, and fat digestibility on day 41 and 42. The enhanced CF digestibility could be linked to the increased gut motility, increased release of energy and nutrients, and the breakdown of fibre during digestion.

Most of the digestibility trials focus on performance and economic considerations, not mainly focusing on N excretions and ammonia emission reduction [57]. As a result, there are no studies solely on the effects of SM on the N excretion of chickens. Feeding SM-containing diets did not modify the dry matter content of the excreta. Since SM is relatively rich in soluble fibre, it presents a potential constraint. According to these results, SM at 20 and 30% does not increase the water content of the excreta. This is positive, since no special exogenous enzymes are available for the degradation of the soluble fibre of SM. At day 41, feeding SM did not affect the N composition of the excreta. On the other hand, the younger chickens excreted less faecal and urinary N, including both NH_4_-N and uric acid N. In birds fed the HFSM30 diet, the uric acid N and the total N excretion was reduced by 18.52% and 15.29%, respectively. It is a positive result from an ammonia emission point of view, since ammonia is formed mainly from the urinary N after bacterial breakdown by urease enzyme [58,59]. It can happen in the barn, during manure storage, or while spreading the manure in the field. The theory behind the decrease is that fibre in the intestine can bind ammonia, reducing the ratio that returns back to the liver and, in this way, lowering uric acid synthesis and excretion. In our findings, urinary N was also reduced by 14 to 22% when SM was fed. However, there is no explanation why this mechanism did not work in the older chickens.

### 4.4. Digesta Viscosity and Short-Chain Fatty Acids

An interesting result of this trial was that the HFSM decreased the viscosity in the jejunal content. The difference in the HFSM30 treatment was significant. Other findings reported that the addition of SM into the broiler-grower diets at a rate of 6% and 8% and into the finisher diets at 10% and 16% significantly increased jejunal and ileal viscosity [48]. This result is against our finding. However, Bilal et al. [48] found that SM at 25% inclusion rate modify fibre digestibility and gut motility. The inclusion of SM at 16% and 20% during the grower and finisher phases, respectively, reduced jejunal viscosity [30], which agrees with the current findings. Therefore, the reason for the lower viscosity at a higher HFSM level could be due to the higher dietary insoluble fibre content. This may enhance gut motility and reduce digesta viscosity. The same tendency was evidenced at day 41, but in this case the differences between the SM treatments and the control group were not significant. The jejunal content viscosity increased with the age. The average viscosity at day 41 was significantly 13.89% higher than that at day 27. The reason for the age-related difference could be that the birds in the finisher phase ate more and, in this way, consumed more soluble dietary fibre, which resulted in increased digesta viscosity in the older chickens.

The jejunal viscosity values show negative correlation with the body-weight gain results. The lower viscosity was found at 30% HFSM level, and the highest BWG belonged also to this treatment. Inclusion of 6% SM during the starter and 10% during the grower or finisher phases had no effect on jejunal digesta viscosity. However, feeding 16% and 20% SM to pullets and layers, respectively [30], as well as inclusion of dehulled SM up to 13.5% measured at 35 days, reduced jejunal digesta viscosity [14], which is not in line with our LFSM results.

Feeding HFSM or LFSM failed to make a difference in the SCFA content and composition of the caecal content. This means, in the frame of SM digestion, there do not arise such fine and soluble particles that enter the caeca.

The results show that the fibre content of the extracted SMs has a significant effect on the production traits, nutrient digestibility, the excretion of the different N compounds, and the gut viscosity of broiler chickens. The SM effects are also age dependent, which should be considered in the diet formulation.

## 5. Conclusions

According to the results of the present study, feeding SM increased the FI of chickens. Although the differences were not significant, the almost 200 g difference in the grower and finisher phase was consequent in the case of HFSM20, HFSM30, and LFSM20 treatments. The BWG was affected only in the finisher period, when the HFSM30 resulted in significantly higher, while the LFSM30 treatment in significantly lower BWG, compared with the control group. SM treatments increased the FCR in all cases, meaning that the increased FI was not in line with the increased BWG. The difference was, however, only significant in the finisher phase for the LFSM20 treatment. In comparison with the control, no treatment effects were found in the carcass composition or the caecal SCFA contents. SM diets improved fat digestion and AMEn but decreased the digestibility of starch. Except in one case, (LFSM30) the N retention of chickens was not affected. Feeding SM resulted in lower urinary and total N excretion in chickens, and the high-fibre form of SM also decreased jejunal viscosity, which was significantly different from the control in the grower phase. In summary, SM can be used as a substitute for soybean meal in broiler diets, but the effects are affected by the fibre content of the product, by the inclusion rate, and also by the age of the animals.

## Figures and Tables

**Table 1:** Measured nutrient content of sunflower meal (%).

Nutrients	HFSM ^1^	LFSM ^2^
Dry matter	90.19	89.93
Crude protein	38.39	43.84
Crude fat	0.52	1.14
Crude fibre	16.68	10.35
Ash	6.73	7.68
Starch	2.45	2.65
Neutral detergent fibre	24.92	20.02
Acid detergent fibre	19.51	14.11
Insoluble dietary fibre	34.12	24.85
Soluble dietary fibre	7.97	8.72
Arginine	0.67	0.70
Isoleucine	0.91	1.10
Lysine	1.72	2.00
Methionine	7.92	9.38
Threonine	2.33	2.72
Valine	2.08	2.15
Cystine	1.00	1.09

^1^ High-fibre sunflower meal, ^2^ Low-fibre sunflower meal.

**Table 2:** Composition of Experimental Diets (g/kg).

Ingredients	Starter	Grower					Finisher				
		C	HFSM 20	HFSM 30	LFSM 20	LFSM 30	C	HFSM 20	HFSM 30	LFSM 20	LFSM 30
Corn	392	403	324	284	401	402	469	386	347	465	459
Wheat	100	100	100	100	100	100	100	100	100	100	100
Extracted Soybean	400	376	221	143	161	53	317	163	85	103	0
HFSM ^1^	55	0	200	300	0	0	0	200	300	0	0
LFSM ^2^	0	0	0	0	200	300	0	0	0	200	300
Sunflower Oil	0	75	108	124	89	95	71	105	121	85	93
MCP ^3^	16	15	15	16	16	16	13	14	14	14	14
Limestone	18	15	13	13	14	13	13	12	11	12	11
Premix ^4^	5	5	5	5	5	5	5	5	5	5	5
Salt	3	3	3	3	3	3	3	3	3	3	3
Sodium Bicarbonate	1	1	1	1	1	1	1	1	1	1	1
Lysine (Biolys)	4	2	5	7	6	8	3	6	8	7	9
Methionine	4	3	3	2	2	2	3	3	2	2	2
Threonine	1	1	1	1	1	1	1	1	2	2	2
Valine	1.0	0.5	0.5	0.5	0.5	0.5	0.5	0.5	0.5	0.5	0.5
Total (g)	1000	1000	1000	1000	1000	1000	1000	1000	1000	1000	1000

C—control diet, ^1^ High-fibre sunflower meal, ^2^ Low-fibre sunflower meal, HFSM20—diet that contains 20% HFSM, HFSM30—diet that contains 30% HFSM, LFSM20—diet that contains 20% LFSM, LFSM30—diet that contains 30% LFSM, ^3^ Monocalcium phosphate; ^4^ Premix was supplied by Agrofeed Ltd. (Győr, Hungary). The active ingredients contained in the premix were as follows (NE per kg of diet): vitamin A—2,000,000 NE, vitamin D3—600,000 NE, vitamin E—6000 mg, menadione—400 mg, thiamine—436 mg, riboflavin—1200 mg, pyridoxin HCl—600 mg, cyanocobalamin—4 mg, niacin—6254 mg, pantothenic acid—1825 mg, folic acid—300 mg, biotin—30 mg, betaine—30,000 mg, BHT—79.5 mg, BHA—79.5 mg, citric acid—71.5 mg, Zn (as ZnO)—8000 mg, Zn (as 3b607)—8000 mg, Cu (as 3b413)—2000 mg, Fe (as FeSO_4_H_2_O)—10,000 mg, Mn (as MnO)—10,000 mg, Mn (as 3b506)—10,000 mg, I [as Ca(IO_3_)2]—300 mg, Se (as C_5_H_11_NO_2_Se)—40 mg, endo-1.4-beta-xylanase—244,000 U, Endo-1.3(4)-beta-glucanase—30,400 U, 6-phytase—100,000 FTU.

**Table 3:** Measured nutrient content of the experimental diets (%).

Nutrients	Starter	Growers					Finishers				
		C	HFSM 20	HFSM 30	LFSM 20	LFSM 30	C	HFSM 20	HFSM 30	LFSM 20	LFSM 30
Dry matter	89.62	89.63	90.58	90.55	89.65	91.31	89.42	89.81	90.01	89.93	89.75
Crude protein	23.28	22.09	21.95	21.95	22.09	21.87	20.94	20.04	19.41	20.09	19.99
Crude fat	7.37	9.05	12.94	13.48	11.43	11.50	9.07	10.23	13.10	9.83	10.33
Crude fibre	2.68	3.40	6.02	7.67	5.53	6.57	3.50	7.16	8.39	4.61	5.57
Ash	6.48	6.41	6.29	6.44	6.55	6.53	6.25	6.20	6.13	6.23	6.34
Calcium	1.06	1.02	1.00	1.04	0.99	1.03	0.92	0.93	0.95	0.91	0.96
Phosphorus	0.61	0.66	0.78	0.85	0.73	0.90	0.62	0.78	0.82	0.77	0.83
Starch	32.16	36.14	30.22	27.70	32.68	30.99	38.16	32.90	30.98	37.61	36.22
GE ^1^ (kJ/g)	17.61	18.08	18.83	19.22	18.86	19.39	17.86	18.81	18.98	18.53	18.57
NDF ^2^	14.99	13.56	15.58	16.32	15.57	16.63	13.79	15.66	16.50	15.91	16.80
Arginine	1.55	1.46	1.52	1.54	1.52	1.54	1.35	1.30	1.27	1.45	1.28
Isoleucine	0.98	0.92	0.91	0.88	0.88	0.83	0.86	0.79	0.74	0.75	0.73
Lysine	1.49	1.25	1.28	1.28	1.27	1.26	1.17	1.13	1.12	1.11	1.08
Methionine	0.65	0.60	0.60	0.59	0.60	0.67	0.56	0.57	0.55	0.58	0.57
Threonine	0.97	0.92	0.93	0.90	0.91	0.89	0.87	0.82	0.90	0.91	0.92
Valine	0.36	0.34	0.35	0.35	0.35	0.35	0.33	0.32	0.32	0.32	0.33

C—control diet, HFSM—High-fibre sunflower meal, LFSM—Low-fibre sunflower meal, HFSM20—diet that contains 20% HFSM, HFSM30—diet that contains 30% HFSM, LFSM20—diet that contains 20% LFSM, LFSM30—diet that contains 30% LFSM, ^1^ Gross energy, ^2^ Neutral detergent fibre.

**Table 4:** The effect of dietary treatments on the production traits (Mean ± SEM).

Parameters	Treatment	Grower Phase (Days 11–24)	Finisher Phase (Days 25–38)	Overall Mean (Days 0–38)
FI ^1^ (g)	C	1084.33 ± 32.08	2100.91 ± 138.46	3185.24 ± 158.23
	HFSM20	1137.93 ± 31.41	2205.36 ± 59.25	3343.28 ± 89.40
	HFSM30	1105.41 ± 20.12	2270.53 ± 22.55	3375.93 ± 36.17
	LFSM20	1152.94 ± 18.45	2162.95 ± 64.83	3315.89 ± 81.92
	LFSM30	1119.34 ± 21.63	2086.25 ± 73.94	3205.59 ± 90.93
	*p*-value	0.375	0.495	0.581
BWG ^2^ (g)	C	858.14 ± 32.65	1547.10 ± 63.96 ^b^	2405.24 ± 90.46
	HFSM20	835.74 ± 24.26	1553.57 ± 29.96 ^b^	2389.31 ± 52.82
	HFSM30	815.14 ± 11.87	1603.41 ± 32.22 ^a^	2418.55 ± 38.29
	LFSM20	848.20 ± 22.81	1430.40 ± 36.39 ^bc^	2278.60 ± 58.42
	LFSM30	824.81 ± 23.34	1389.32 ± 42.80 ^c^	2214.13 ± 62.30
	*p*-value	0.719	0.023	0.125
FCR ^3^ (g/g)	C	1.27 ± 0.01 ^b^	1.36 ± 0.07 ^b^	1.32 ± 0.04 ^b^
	HFSM20	1.36 ± 0.02 ^a^	1.42 ± 0.02 ^ab^	1.40 ± 0.02 ^ab^
	HFSM30	1.36 ± 0.01 ^a^	1.42 ± 0.02 ^ab^	1.40 ± 0.01 ^ab^
	LFSM20	1.36 ± 0.02 ^a^	1.51 ± 0.03 ^a^	1.46 ± 0.02 ^a^
	LFSM30	1.36 ± 0.02 ^a^	1.50 ± 0.02 ^ab^	1.45 ± 0.01 ^a^
	*p*-value	0.001	0.036	0.008

C—control diet, HFSM—high-fibre sunflower meal, LFSM—low-fibre sunflower meal, HFSM20—diet that contain 20% HFSM, HFSM30—diet that contain 30% HFSM, LFSM20—diet that contain 20% low-fibre sunflower meal, LFSM30—diet that contain 30% LFSM, ^1^ Feed intake, ^2^ Body weight gain, ^3^ Feed conversion ratio, ^a,b,c^ means with different superscripts in the same column are significantly different (*p* < 0.05).

**Table 5:** Effect of dietary treatments on carcass composition (day 43) (Mean ± SEM, *n* = 8 birds per treatment).

Treatments	Carcass (%)	Breast Meat (%)	Thigh (%)	Abdominal Fat (%)
C	67.89 ± 0.76	23.08 ± 0.66	19.42 ± 0.47	0.83 ± 0.11 ^ab^
HFSM20	67.19 ± 0.63	22.43 ± 0.81	19.51 ± 0.54	0.70 ± 0.10 ^ab^
HFSM30	66.96 ± 0.61	21.66 ± 0.57	19.32 ± 0.56	0.64 ± 0.11 ^b^
LFSM20	63.70 ± 2.93	21.91 ± 0.64	18.71 ± 0.41	1.18 ± 0.19 ^a^
LFSM30	65.13 ± 1.48	20.08 ± 0.95	18.47 ± 0.35	0.95 ± 0.08 ^ab^
*p*-value	0.289	0.083	0.479	0.030

C—control diet, HFSM—high-fibre sunflower meal, LFSM—low-fibre sunflower meal, HFSM20—diet that contain 20% HFSM, HFSM30—diet that contain 30% HFSM, LFSM20—diet that contain 20% low-fibre sunflower meal, LFSM30—diet that contain 30% LFSM, ^a,b^ means with different superscripts in the same column are significantly different (*p* < 0.05).

**Table 6:** Faecal nutrient digestibility and nitrogen retention (Mean ± SEM).

Treatments	Fat (%)	Starch (%)	AME ^1^ (kJ/g)	AMEn ^2^ (kJ/g)	N Retention (%)
Day 27					
C	91.3 ± 0.24 ^b^	85.2 ± 0.26 ^a^	13.9 ± 0.12 ^c^	13.7 ± 0.12 ^c^	63.1 ± 1.16 ^b^
HFSM20	92.0 ± 0.50 ^abc^	82.1 ± 0.36 ^bc^	13.7 ± 0.04 ^c^	13.5 ± 0.04 ^c^	64.7 ± 0.81 ^b^
HFSM30	90.5 ± 0.50 ^bc^	79.0 ± 0.27 ^d^	13.9 ± 0.06 ^c^	13.7 ± 0.06 ^c^	63.6 ± 1.02 ^b^
LFSM20	93.5 ± 0.16 ^a^	83.0 ± 0.17 ^b^	14.5 ± 0.08 ^b^	14.3 ± 0.08 ^b^	66.1 ± 0.66 ^ab^
LFSM30	93.7 ± 0.34 ^a^	81.4 ± 0.32 ^c^	15.2 ± 0.12 ^a^	15.0 ± 0.12 ^a^	69.4 ± 0.96 ^a^
*p*-value	<0.001	<0.001	<0.001	<0.001	<0.001
Day 41					
C	88.1 ± 0.53 ^b^	82.9 ± 0.62 ^a^	13.4 ± 0.30 ^bc^	13.2 ± 0.30 ^bc^	64.0 ± 1.52
HFSM20	84.4 ± 0.42 ^c^	79.9 ± 0.74 ^bc^	13.5 ± 0.32 ^bc^	13.3± 0.32 ^bc^	63.6 ± 1.24
HFSM30	87.5 ± 0.51 ^b^	76.3 ± 0.43 ^d^	13.4 ± 0.55 ^c^	13.1 ± 0.55 ^c^	62.3 ± 1.50
LFSM20	88.5 ± 0.64 ^b^	81.3 ± 0.61 ^ab^	14.0 ± 0.47 ^ab^	13.8 ± 0.46 ^ab^	66.9 ± 1.61
LFSM30	92.2 ± 0.42 ^a^	78.3 ± 0.60 ^cd^	14.2 ± 0.44 ^a^	13.9 ± 0.42 ^a^	65.7 ± 2.83
*p*-value	<0.001	<0.001	<0.001	<0.001	0.424

C—control diet, HFSM—high-fibre sunflower meal, LFSM—low-fibre sunflower meal, HFSM20—diet that contain 20% HFSM, HFSM30—diet that contain 30% HFSM, LFSM20—diet that contain 20% low-fibre sunflower meal, LFSM30—diet that contain 30% LFSM, ^a,b,c,d^ means with different superscripts in the same column are significantly different (*p* < 0.05). ^1^ apparent metabolisable energy, ^2^ apparent metabolisable energy nitrogen corrected.

**Table 7:** The dry matter content and the different nitrogen forms of the excreta (% of fresh excreta samples) (Mean ± SEM).

Age	Treatment	Faecal DM	NH_4_-N	Uric Acid-N	Urinary-N	Faecal-N	Total N
Day 27	C	20.62 ± 0.41	0.10 ± 0.00 ^a^	0.27 ± 0.01 ^a^	0.37 ± 0.01 ^a^	0.48 ± 0.03	0.85 ± 0.04 ^a^
	HFSM20	21.21 ± 0.59	0.08 ± 0.01 ^bc^	0.24 ± 0.01 ^ab^	0.31 ± 0.01 ^b^	0.42 ± 0.02	0.73 ± 0.03 ^ab^
	HFSM30	20.65 ± 0.60	0.08 ± 0.00 ^bc^	0.22 ± 0.01 ^b^	0.29 ± 0.01 ^b^	0.43 ± 0.02	0.72 ± 0.02 ^b^
	LFSM20	20.98 ± 0.25	0.07 ± 0.01 ^c^	0.24 ± 0.00 ^ab^	0.31 ± 0.01 ^b^	0.44 ± 0.01	0.75 ± 0.02 ^ab^
	LFSM30	20.77 ± 0.32	0.09 ± 0.01 ^ab^	0.23 ± 0.02 ^b^	0.32 ± 0.02 ^ab^	0.40 ± 0.02	0.72 ± 0.04 ^b^
	*p*-value	0.879	0.001	0.004	0.002	0.121	0.028
Day 41	C	18.52 ± 0.50	0.08 ± 0.00	0.21 ± 0.01	0.30 ± 0.01	0.38 ± 0.02	0.68 ± 0.03
	HFSM20	19.05 ± 0.56	0.08 ± 0.00	0.19 ± 0.01	0.28 ± 0.01	0.32 ± 0.01	0.60 ± 0.02
	HFSM30	19.46 ± 0.48	0.08 ± 0.00	0.19 ± 0.00	0.26 ± 0.01	0.32 ± 0.01	0.59 ± 0.02
	LFSM20	18.27 ± 0.58	0.09 ± 0.00	0.20 ± 0.01	0.29 ± 0.01	0.34 ± 0.02	0.63 ± 0.03
	LFSM30	18.65 ± 0.49	0.08 ± 0.00	0.21 ± 0.01	0.29 ± 0.02	0.33 ± 0.02	0.62 ± 0.04
	*p*-value	0.646	0.430	0.222	0.324	0.118	0.156

C—control diet, HFSM—high-fibre sunflower meal, LFSM—low-fibre sunflower meal, HFSM20—diet that contain 20% HFSM, HFSM30—diet that contain 30% HFSM, LFSM20—diet that contain 20% low-fibre sunflower meal, LFSM30—diet that contain 30% LFSM, ^a,b,c^ means with different superscripts in the same column are significantly different (*p* < 0.05).

**Table 8:** Feed and age interaction effect of the jejunal digesta viscosity (cP) (Mean ± SEM, *n*, 74).

Age	Feed	Viscosity
Day 27	C	4.0 ± 0.12 ^a^
	HFSM20	3.7 ± 0.17 ^ab^
	HFSM30	3.2 ± 0.07 ^b^
	LFSM20	3.7 ± 0.11 ^ab^
	LFSM30	3.5 ± 0.17 ^ab^
*p*-value		<0.001
Day 41	C	4.0 ± 0.08 ^ab^
	HFSM20	4.2 ± 0.16 ^a^
	HFSM30	3.4 ± 0.03 ^b^
	LFSM20	4.4 ± 0.14 ^a^
	LFSM30	4.3 ± 0.17 ^a^
*p*-value		<0.001
SEM		0.06
Feed	C	4. 0 ± 0.09 ^a^
	HFSM20	4.0 ± 0. 09 ^a^
	HFSM30	3.3 ± 0. 09 ^b^
	LFSM20	4.0 ± 0. 09 ^a^
	LFSM30	3.9 ± 0.11 ^a^
Age	Day 27	3.6 ± 0.06 ^b^
	Day 41	4.1 ± 0.06 ^a^
*p*-values	Feed	<0.001
	Age	<0.001
	Feed X age	0.005

C—control diet, HFSM–high-fibre sunflower meal, LFSM—low-fibre sunflower meal, HFSM20—diet that contain 20% HFSM, HFSM30—diet that contain 30% HFSM, LFSM20—diet that contain 20% low-fibre sunflower meal, LFSM30—diet that contain 30% LFSM, ^a,b^ means with different letters are significantly different (*p* < 0.05).

**Table 9:** The effect of dietary treatments on the caecal short-chain fatty acids of broiler chicks (Mean ± SEM).

Age	Treatments	Acetic Acid	Propionic Acid	Isobutyric Acid	Butyric Acid	Isovaleric Acid	Valeric Acid
Day 27	C	2.28 ± 0.19	0.52 ±0.07	0.07 ± 0.01	0.50 ± 0.07	0.07 ± 0.02	0.07 ± 0.02
	HFSM20	2.16 ± 0.24	0.51 ± 0.06	0.06 ± 0.01	0.43 ± 0.06	0.08 ± 0.02	0.08 ± 0.01
	HFSM30	2.16 ± 0.25	0.49 ± 0.06	0.07 ± 0.01	0.46 ± 0.06	0.08 ± 0.02	0.07 ± 0.01
	LFSM20	2.43 ± 0.24	0.50 ± 0.06	0.06 ± 0.01	0.50 ± 0.07	0.06 ± 0.01	0.09 ± 0.01
	LFSM30	2.33 ± 0.24	0.43 ± 0.06	0.06 ±0.01	0.57 ± 0.07	0.07 ± 0.01	0.08 ± 0.01
	*p*-value	0.918	0.888	0.974	0.692	0.816	0.938
Day 41	C	2.23 ± 0.23	0.50 ± 0.06	0.06 ± 0.01	0.40 ± 0.04	0.07 ± 0.01	0.06 ± 0.01
	HFSM20	2.47 ± 0.20	0.52 ± 0.06	0.06 ± 0.01	0.47 ± 0.06	0.07 ± 0.01	0.07 ± 0.02
	HFSM30	2.13 ± 0.24	0.50 ± 0.09	0.04 ± 0.01	0.46 ± 0.07	0.06 ± 0.01	0.05 ± 0.01
	LFSM20	2.26 ± 0.26	0.48 ± 0.06	0.06 ± 0.01	0.51 ± 0.06	0.07 ± 0.01	0.06 ± 0.01
	LFSM30	2.30 ± 0.26	0.49 ± 0.06	0.06 ± 0.01	0.48 ± 0.05	0.07 ± 0.01	0.07 ± 0.01
	*p*-value	0.903	0.991	0.488	0.724	0.974	0.553

C—control diet, HFSM–high-fibre sunflower meal, LFSM—low-fibre sunflower meal, HFSM20—diet that contain 20% HFSM, HFSM30—diet that contain 30% HFSM, LFSM20—diet that contain 20% low-fibre sunflower meal, LFSM30—diet that contain 30% LFSM.

## Data Availability

All data generated or analysed during this study are included in this published article.
